# Effects of maze appearance on maze solving

**DOI:** 10.3758/s13414-024-03000-7

**Published:** 2025-01-10

**Authors:** Yelda Semizer, Dian Yu, Qianqian Wan, Benjamin Balas, Ruth Rosenholtz

**Affiliations:** 1 Department of Humanities and Social Sciences, New Jersey Institute of Technology, Newark, NJ USA; 2 Computer Science and Artificial Intelligence Laboratory, Massachusetts Institute of Technology, Cambridge, USA; 3 Department of Psychology, The Ohio State University, Columbus, OH USA; 4 Department of Psychology, North Dakota State University, Fargo, ND USA; 5 Department of Brain and Cognitive Sciences, Massachusetts Institute of Technology, Cambridge, USA

**Keywords:** Visual perception, 2D shape and form, Spatial vision

## Abstract

As mazes are typically complex, cluttered stimuli, solving them is likely limited by visual crowding. Thus, several aspects of the appearance of the maze – the thickness, spacing, and curvature of the paths, as well as the texture of both paths and walls – likely influence the performance. In the current study, we investigate the effects of perceptual aspects of maze design on maze-solving performance to understand the role of crowding and visual complexity. We conducted two experiments using a set of controlled stimuli to examine the effects of path and wall thickness, as well as the style of rendering used for both paths and walls. Experiment 1 finds that maze-solving time increases with thicker paths (thus thinner walls). Experiment 2 replicates this finding while also showing that maze-solving time increases when mazes have wavy walls, which are likely more crowded, rather than straight walls. Our findings imply a role of both crowding and figure/ground segmentation in mental maze solving and suggest reformulating the growth cone models.

## Introduction

As early as elementary school, children can solve pen and paper mazes (Krikorian & Bartok, 1998), tracing a path through the corridors from start to finish without crossing any walls. Mazes, broadly construed, potentially provide a rich paradigm for studying such problems as eye movement planning, visual perception of complex stimuli, visual development, solving of multi-step visual cognition problems, and combining information from fovea and periphery and across multiple fixations. While children’s mazes are not very natural stimuli, they are related to real-world stimuli and problems, such as tracing a path in a subway map, or integrating a contour to untangle a garden hose. Furthermore, one can relatively easily control the appearance of the stimuli, varying path complexity, curvature, and the appearance of paths and walls. Mazes are fun, and one can easily recruit subjects across a wide age range. All of these factors make maze solving an interesting paradigm for studying vision.

**Fig. 1:**
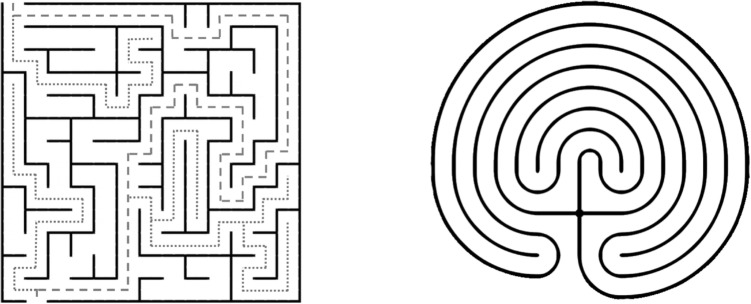
Example mazes. The maze on the left has multiple long dead ends (indicated by *dotted lines*) which potentially increases the solving time and the memory load because the solver needs to remember where they were while backtracking a path, which makes the maze cognitively difficult. The maze on the right has a single path but the path is curved and the perceptual similarity between its corridors can make it difficult to keep track of one’s location on the path

With the possible exception of some very easy mazes for small children, mental maze solving – i.e., imagining the path rather than marking it on the paper – tends to require more than a momentary glance; maze solving is typically a multistep process, and mazes can be difficult for different reasons. One can start from the entrance and follow the path until encountering an intersection. At the intersection, the person needs to make a decision as to which direction to turn, and follow the chosen path until reaching a dead end or an exit. More branches in the path make the task more difficult for cognitive reasons: more paths to explore, and more memory load due to the need to remember previously eliminated paths. However, perceptual factors also affect difficulty; even a maze with a single corridor leading directly from start to finish could be perceptually difficult (Fig. 1).

A number of algorithmic strategies for solving mazes have been proposed, including but not limited to random mouse, wall follower, and Trémaux’s algorithm (Abelson & Disessa, 1981; Sadik et al., 2010). The most “naive”, and highly inefficient, strategy would be to randomly choose a path at each intersection until one of them leads to the exit, while always moving forward; this is known as the “random mouse”. Another strategy is consistently to follow the right or left wall throughout the maze, also known as the “wall follower”. Another more generalizable strategy uses Trémaux’s algorithm, which requires picking paths randomly at intersections, marking paths that have been visited, and backtracking dead ends to find the correct solution.

These strategies, however, are more what a computer might do, making little use of the strengths of human vision. They effectively require vision only within a small foveal region; one must merely use vision to stay on the path, and to discern the existence of local intersections. Human vision can, in principle, provide at least some information about the maze as a whole. When fixating a given point on the path, one may perceive the exit, and perhaps some of the path near to it. One can probably see somewhat ahead on the path, not to mention portions of the maze one has recently visited. Seeing the shape of the path ahead may allow one to make longer saccades to more distant parts of the maze. One may see an extended clear path somewhere in the maze, and perhaps try to navigate towards that path. This information, though almost certainly imperfect and limited, would likely make maze-solving more efficient than the brute-force strategies mentioned above. Gathering this information from across the field of view naturally makes use of peripheral vision.

Mental maze solving is in some sense equivalent to many of the visual cognition tasks of Ullman (1996), Jolicoeur et al. (1991), and Jolicoeur and Ingleton (1991). Asking observers whether two points lie on the same or different curves essentially asks whether a path exists between the two points, where essentially each curve is like a maze “corridor”. Asking observers whether a given point lies inside or outside of a closed figure could be reframed as asking whether a clear path lies between the point and an arbitrary point clearly inside the figure. For the purposes of this paper, we will refer to a number of these tasks as maze-solving tasks, though we will occasionally refer instead to the specific task in discussing previous work.

Researchers have found evidence for the use of peripheral vision in maze-solving and related tasks. Jolicoeur et al. (1986) first found that reaction time on a curve following task depends on the length of the path. This dependence on path length could be consistent with either a strategy that uses only very local, foveal information to follow the path or with using peripheral vision plus eye movements to perform the task. However, they next effectively forced observers to maintain fixation while performing the task, by reducing the display time (Jolicoeur et al., 1986). Observers could largely still perform the task while fixating, though they made more errors and took longer when solving mazes with longer path lengths. Researchers have interpreted these results to suggest that attention or processing needs time to spread along the path (Jolicoeur et al., 1991; Roelfsema, 2006). The present paper is agnostic about the role of spread of attention, and instead focuses on perceptual aspects of peripheral vision. The fact that people can do the task while fixating suggests that observers are capable of using peripheral vision to solve a mental path-following task.

Furthermore, researchers have studied eye movements during mental maze solving. Crowe et al. (2000) asked observers to mentally solve 2D rectangular mazes presented on a computer screen. Participants indicated with a key press which of the possible exits connected to the starting point. In addition to path length, they found that the number of turns (i.e., 90 degree change in the direction of the path) influenced solution time, with observers taking longer to solve mazes with more turns. In addition, they found that fixation duration depended on both the length of the upcoming saccade, and on the number of turns in the path between the two fixations. If observers solved the mazes using only foveal information, one would not expect longer fixation durations as a function of the characteristics of later parts of the path. Rather, these findings suggest that observers use their peripheral vision to help solve a maze.

Although this prior work suggests a role for peripheral vision in mental maze solving, the mechanisms by which peripheral vision limits the task performance require more research. One possible mechanism is visual “crowding", a main limiting factor in peripheral vision (Rosenholtz, 2016). Crowding refers to the difficulty performing peripheral tasks in the presence of clutter (Bouma, 1970; Levi, 2008; Pelli et al., 2007, 2004). The well-known effects of reduced peripheral acuity have only a modest effect when compared with crowding (Rosenholtz, 2016). Early behavioral work demonstrated difficulty identifying a peripheral letter flanked by other letters when the flankers fell within a *critical spacing* of roughly 0.4 to 0.5 times the eccentricity, or distance to point of fixation (Bouma, 1970). Crowding not only interferes with identification, it can lead to mislocalization of details (e.g., Korte, 1923), and has been described as leading peripheral stimuli to “lose the quality of ‘form’ ” Lettvin (1976). Critically, the degree of crowding depends not merely on the spacing between elements of the stimulus. Rather, researchers have found a complex dependence on the specific details of the stimulus, such as its perceptual organization (e.g., just a few examples: (Banks & White, 1984; Kooi et al., 1994; Manassi et al., 2012)). Crowding depends a lot on the specifics of the stimuli (Pelli et al., 2006; Pelli & Tillman, 2008). More complex stimuli, such as letters with more strokes, often lead to more crowding. Crowding can also occur in the fovea (Levi et al., 2002), leading, for instance, to difficulty distinguishing between five and six closely spaced vertical lines. Foveal crowding may well add to the difficulty solving particularly dense mazes, but is not likely a factor in those studied here.

Crowding is likely relevant for mental maze solving. Mazes are often quite cluttered – paths are closely flanked by other paths, for instance – and crowding might limit how far along the path one could clearly see at a glance. More crowded mazes might require more and/or longer fixations to solve the maze. Crowded mazes mean that many details of their appearance may affect performance, even if they have the same underlying topology, i.e., the same path lengths and number and location of branches.

**Fig. 2:**
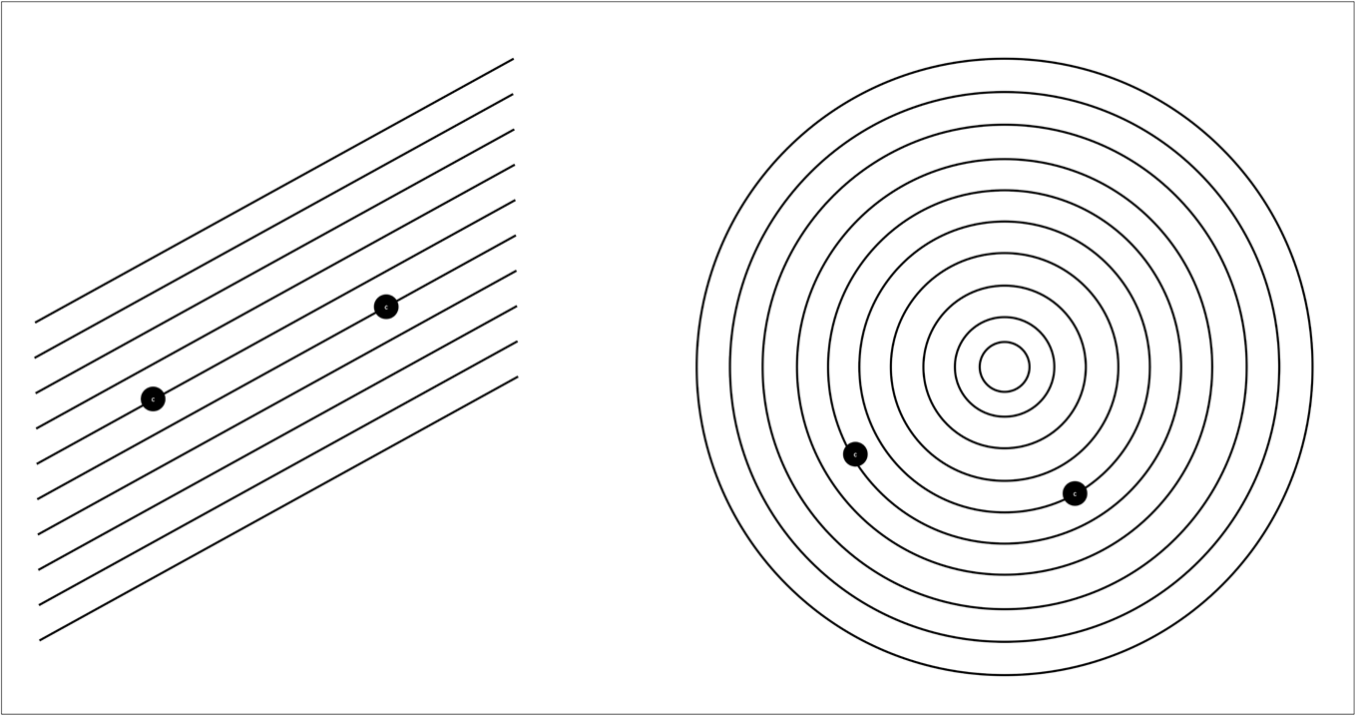
Deciding whether two points lie on the same line or not is easier than deciding whether they lie on the same curve, although the path distance is roughly the same between the two points. Also, curve tracing is slower when lines are closer together, and when they are more curved. Based on stimuli from Jolicoeur et al. (1991)

If crowding were a factor in maze solving, we would expect effects of both the density of paths and their complexity. For example, more curved paths might be more complex, leading to poorer peripheral vision and longer solving times. In fact, Jolicoeur et al. (1991) asked observers to judge whether two points lay on the same curve. Their stimuli ranged from straight parallel lines through curved lines nested in an onion-like fashion (Fig. 2). They found longer reaction times for more closely spaced curves, and for higher curvature. Even for their simple displays, appearance matters.

Additionally, if peripheral crowding were a factor in maze solving, we would also expect performance to be fairly scale-invariant, i.e., invariant to viewing distance. This scale invariance results from both the critical spacing of crowding (Bouma, 1970; Pelli et al., 2004) and acuity varying linearly with eccentricity (Van Essen & Anderson, 1995). Several studies have shown scale invariance in maze-solving and related tasks (Crowe et al., 2000; Jolicoeur & Ingleton, 1991).

**Fig. 3:**
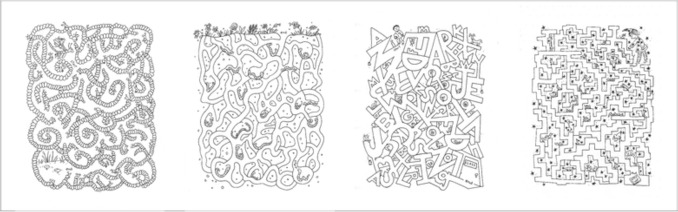
Examples of kids mazes from a book of children’s mazes (“Maze Mania", Woodworth (2005)). Published with permission from Dover Publications

In fact, Jolicoeur et al. (1991) suggested that peripheral crowding might play a role in their visual cognition tasks, though they referred to this as “lateral masking”, as was common at the time. Researchers had not yet developed models of crowding; as a result, Jolicoeur and colleagues focused on crowding’s dependence on spacing, in a task-specific model. In particular, they suggest that curve tracing utilizes a shifting “beam” or processing region, of adjustable size.^1^ The beam must contain a single, mostly straight path; if it contains more than one path, or too sharp a turn, it must shrink in size until the conditions are satisfied. Larger beams allow for faster travel along the path. This would predict their effects of both path density and path curvature (i.e., longer reaction times for denser curves and higher curvature). Because doubling the size of a stimulus would double the allowable size of the shifting beams, this would also explain the scale invariance just discussed (Pooresmaeili & Roelfsema, 2014). However, this model does not address the basic eccentricity dependence of crowding; that is, that the effect of crowding is larger at greater eccentricities.

Roelfsema and colleagues (e.g., Jeurissen et al., 2016; Pooresmaeili and Roelfsema, 2014) further developed Jolicoeur et al. (1991)’s model into their “growth cone” model, in which beams or “growth cones” must grow with eccentricity, due to cortical magnification. As a result, vision may be unable to process more eccentric regions without an eye movement, due to crowding. As with the shifting beams of Jolicoeur et al. (1991)’s model, larger growth cones support faster maze solving due to allowing faster travel along the path.

**Fig. 4:**
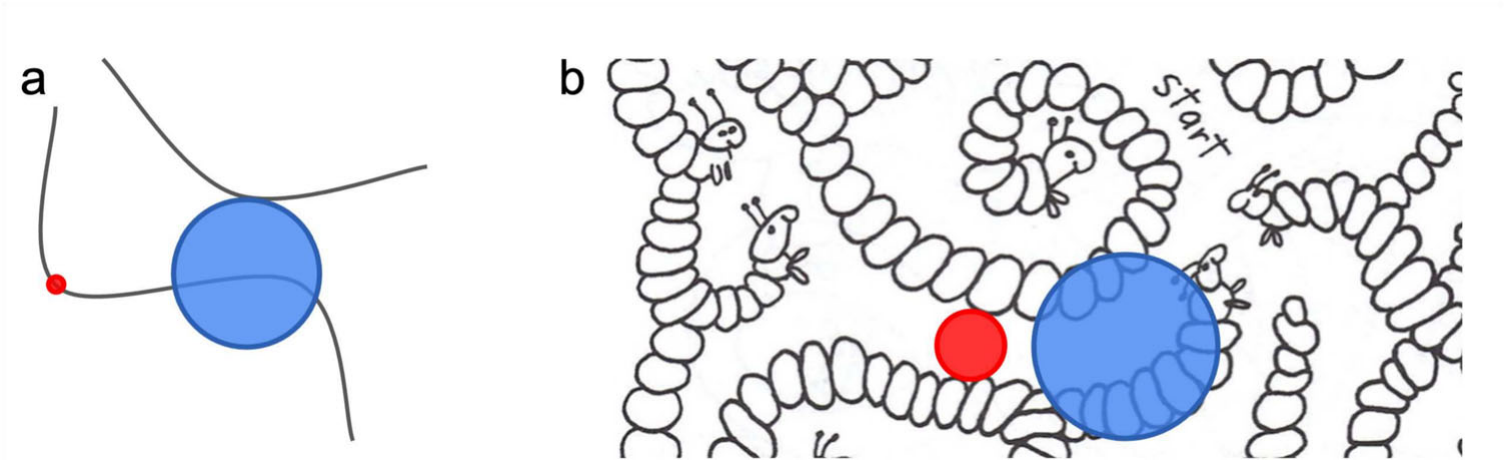
**a** In the growth cone model of Pooresmaeili and Roelfsema (2014), growth cones centered on the current path extend until reaching any neighboring path. The *blue circle* shows an example. **b** By this description, an equivalent growth cone for a complex maze (“Maze Mania", Woodworth (2005)) might include clutter from the maze walls (*blue circle*). A simple alternative for complex mazes would include only the path, as indicated by the *red circle*. The equivalent for the stimulus in **a**, however, would remain the same small size regardless of the path curvature or spacing of neighboring paths (*red circle*). A more complex alternative might instead depend on the stimulus, and in particular on how easily one can see the path forward given factors such as peripheral crowding. Published with permission from Dover Publications

Both the growth cone model and its precursor address visual crowding primarily in terms of path spacing. These models essentially treat crowding like biased competition (Desimone & Duncan, 1995), in which having more than one thing (e.g., one path) within the processing region prohibits performing the task. However, not only does crowding depend on stimulus factors other than space, but even under conditions of crowding one can perceive rather a lot about the stimulus – the presence of clutter does not render an observer incapable of making any judgments about the stimulus (for a review, see Rosenholtz (2016)). If crowding is a critical factor, the characteristics of the stimulus should matter. The novelty of our study is that we consider the role of stimulus elements – i.e., the maze appearance – beyond spacing of paths, path length, and path curvature or number or turns.

If more complex aspects of maze appearance affect maze-solving performance, then this would illuminate a limitation of these prior models of maze solving. Mazes can differ greatly in their appearance, due to creative ways of rendering the walls and the paths (see Fig. 3), even though their underlying topologies are similar, i.e., path lengths, number of branches, etc. Crowding’s dependence on stimulus factors would predict that such mazes could lead to very different performance. How does one generalize the models of Jolicoeur et al. (1991) and Pooresmaeili and Roelfsema (2014) to such complex stimuli?

The models of Jolicoeur et al. (1991) and Pooresmaeili and Roelfsema (2014), suggest path width or spacing as an important factor in maze solving. These models have been applied to stimuli in which the path consists of a thin line, and the “walls” consist simply of white space between the paths. In our understanding, the growth cone for these stimuli covers the distance between flanking paths; i.e., in their stimuli, between neighboring thin lines (Fig. 4a, blue circle). This might be plausible for such simple stimuli. However, for the mazes in Fig. 3, such a growth cone would contain the complex wall stimuli (Fig. 4b, blue circle). Such a growth cone would contain a good deal of clutter, seemingly not in the spirit of the model (not to mention being a bit ill-defined for some of the mazes). A simple alternative for complex mazes would be that the growth cone instead extends to fill the relatively uncluttered path region, with a maximum size such that it just touches the walls (Fig. 4, red circle). A more complex alternative might be that the size of the growth cone depends on the characteristics of the stimulus, and in particular on how easily one can see the path forward given factors such as peripheral crowding.

The present study investigates the effects of several aspects of maze design on maze-solving performance. Current understanding of crowding suggests that each could have an impact on the observer’s ability to perceive a clear path in the periphery, thus affecting the time and number of fixations needed to solve a given maze. Our goal is to gain a more comprehensive understanding of how to update prior models to address more complex stimuli and to a deeper understanding of crowding. We fix the path structure of simple mazes, and test the effects of specific manipulations in appearance. Experiment 1 covaries path and wall thickness; thicker paths mean thinner walls between paths, for the same size mazes. Line thickness has been shown to have an impact on peripheral crowding in a somewhat complex way; changing the thickness of lines can make it either easier or harder to distinguish between crowded peripheral items, depending on the exact details of the stimulus, and this change in discriminability affects visual search (Chang & Rosenholtz, 2016). Moreover, as we will discuss, it is unclear how the aforementioned models generalize to a change in wall thickness. As a result, we expect an effect of path vs. wall thickness but neither previous results from crowding and nor maze solving clearly indicate the likely direction of the effect. Experiment 2 varies maze crowding by varying the waviness of the maze walls as well as their thickness. Wavy, more complex walls should increase the effect of crowding and result in longer reaction times.

**Fig. 5:**
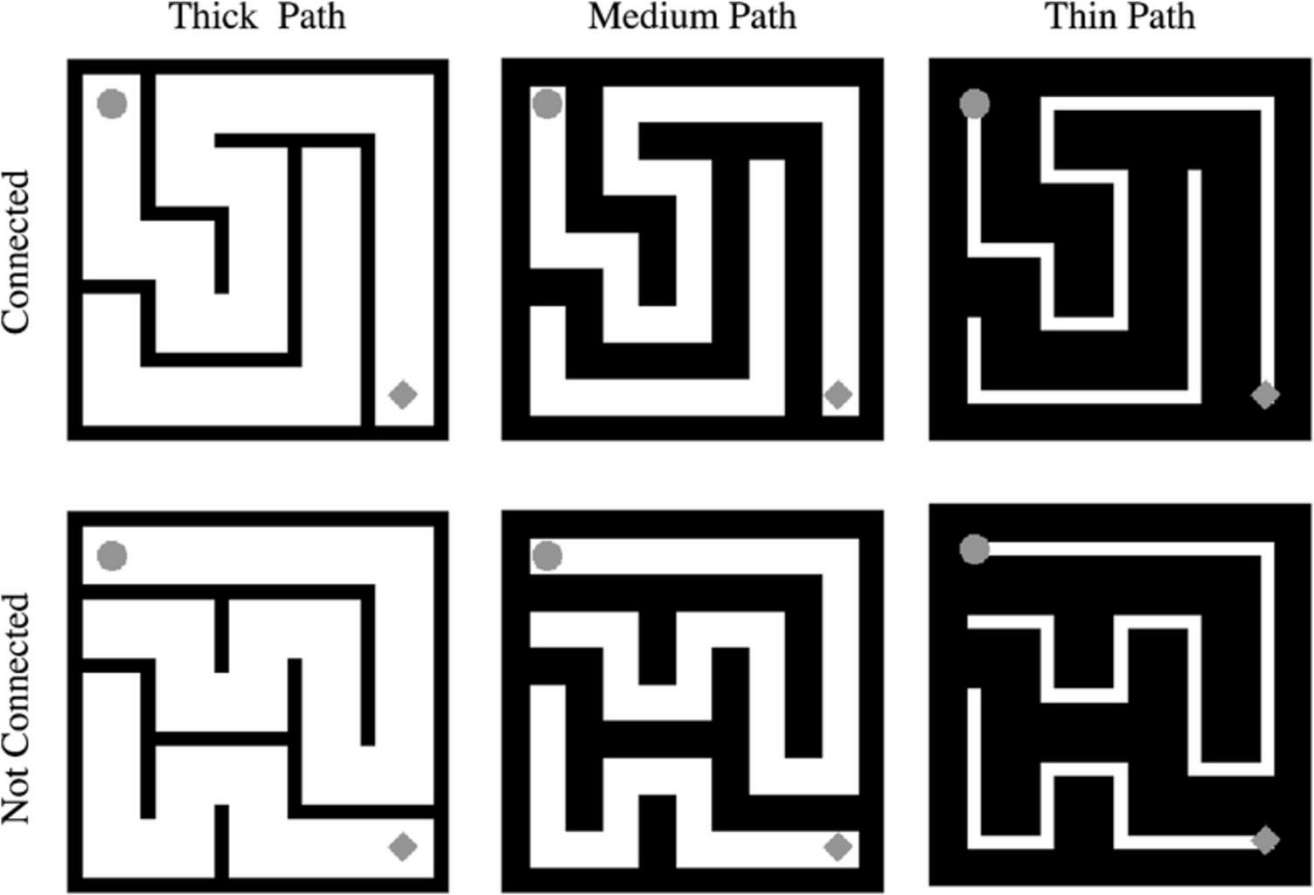
Example mazes used as stimuli in Experiment 2. *Columns left to right* show mazes with thick paths, medium paths, and thin paths. *Rows top to bottom* show mazes with connected paths (top left and bottom right cells, marked by a *dot* and a *diamond*, are connected) and mazes with disconnected paths

## Experiment 1

### Methods

#### Observers

A total of 24 observers participated in the experiment. All were naïve to the purpose of the experiment and received compensation for their participation. All observers self-reported normal or corrected-to-normal visual acuity.

#### Apparatus

Stimuli were presented on a 27-in Apple monitor with a refresh rate of 60 Hz. The resolution was set to $2048 \times 1152$ pixels. Observers viewed the monitor at a distance of 70 cm with their head position constrained by a chin rest.^2^ All stimulus presentation and response collection routines were implemented using PsychoPy (Peirce, 2007).

#### Stimuli

We generated 30 mazes, each with dimensions of $5 \times 5$, using a maze generation algorithm in Python (rosettacode.org/wiki/Maze_generation) with modifications to make it more appropriate for our experimental task (Fig. 5). Each maze extended $8^{\circ } \times 8^{\circ }$ of visual angle. Half of the mazes had a path connecting the top left and bottom right cell, marked by a dot and a diamond, respectively. The other half did not have a path connecting the top left and bottom right cell.

We then manipulated the thickness of the path in each maze. The path width was set to $0.3^{\circ }$, $0.8^{\circ }$, and $1.3^{\circ }$ of visual angle in the thin, medium, and thick path conditions, respectively. This manipulation resulted in a total of 90 mazes. Each maze was then flipped $180^{\circ }$ to create another maze, resulting a total of 180 mazes in our stimuli set.

#### Procedure

Each trial began with a fixation cross presented at the center of the screen for 500 ms. The background was set to uniform gray. Once the trial was initiated by a key press, a maze was presented at center of the screen. We asked observers to indicate whether there was a path connecting the dot and diamond located in the top left and bottom right corners of the maze by pressing one of the up or down arrows. The maze remained on the screen until the observer made a response. Observers were allowed to move their eyes but were asked to respond as quickly and as accurately as possible.

**Fig. 6:**
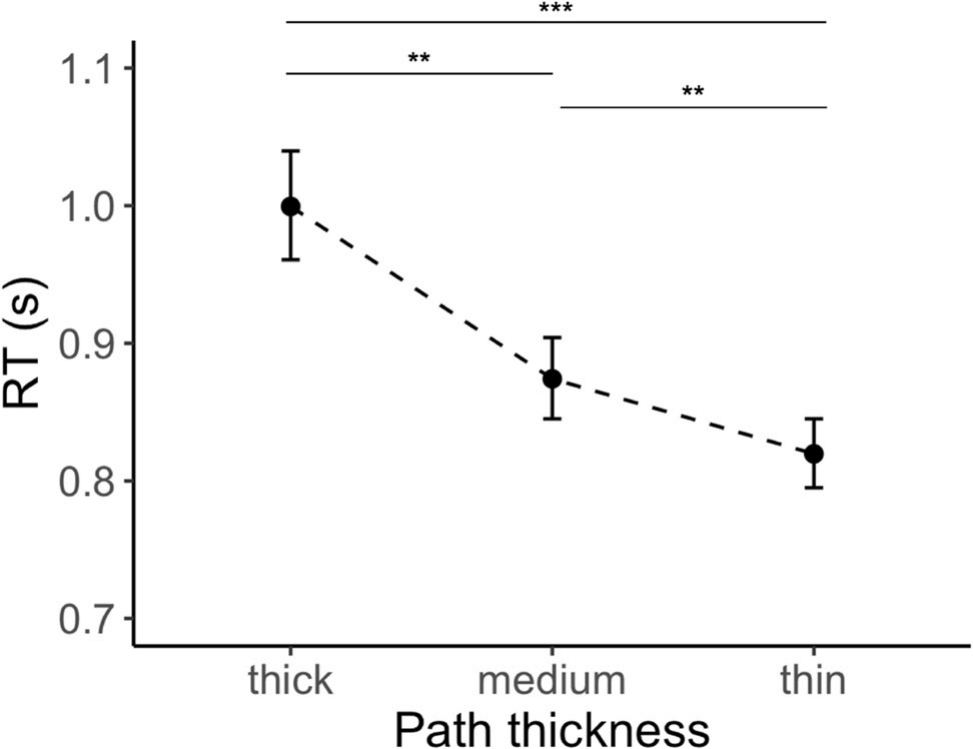
Average response times (s) in thick, medium, and thin path conditions (back-transformed from log for easier interpretation). As the thickness of the path decreases, so does the response time. *Error bars* indicate standard error of the mean. **$p<0.01$, ***$p<0.001$

**Fig. 7:**
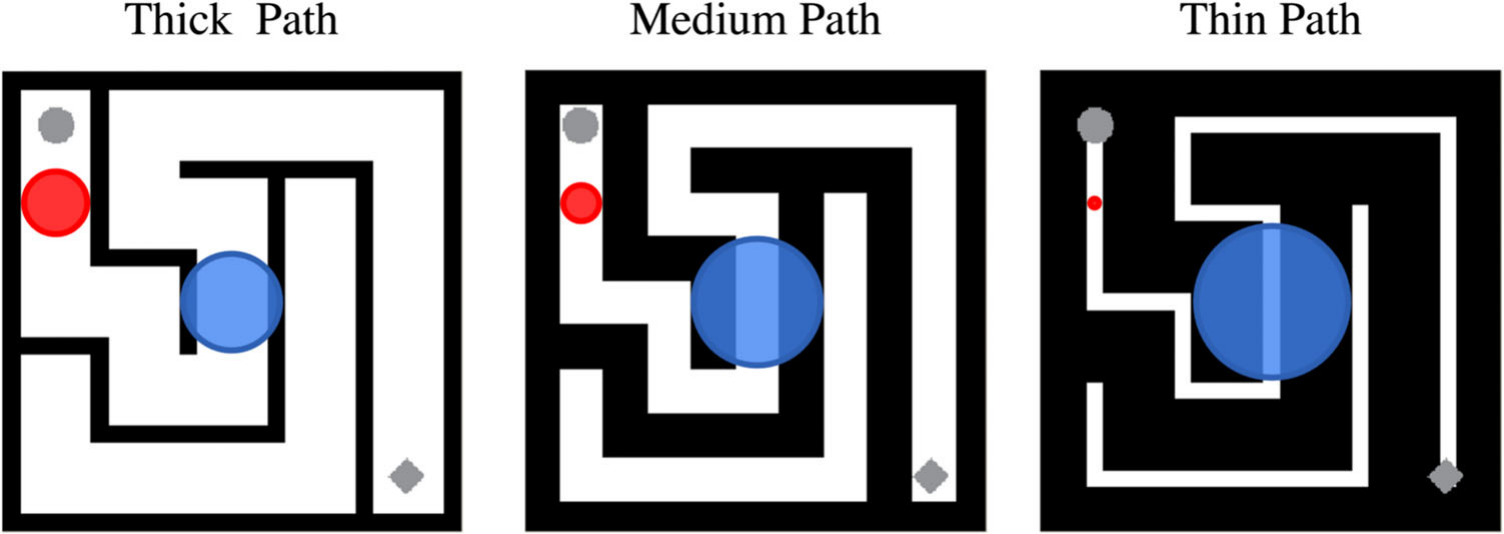
Two interpretations of the growth cone model (color coding as in Fig. 4) on sample stimuli from Experiment 2. The size of the* blue circle* correlates with the data (i.e., faster solving time for thinner paths) while the size of the *red circle* does not

The order of the mazes presented in each trial was randomized for each observer. Trials were blocked by the wall thickness. Each participant completed six blocks with 30 trials in each block, resulting in a total of 180 trials. The block sequence was semi-randomized across participants.

### Results

#### Response time

We analyzed log-transformed response times for correct trials. Figure 6 shows the mean of these response times in the thick, medium, and thin path conditions (back-transformed from log for easier interpretation). A one-way repeated measures ANOVA revealed a significant effect of path thickness on response times, $F(2,46)=29.40,p<0.001, \eta ^2=0.56$. Post-hoc pairwise comparisons with Bonferroni adjustments showed significant differences between groups. Observers were significantly slower in solving mazes with thick paths ($M=1.00,SE=0.04$) compared to mazes with medium paths ($M=0.87,SE=0.03$), with $p<0.01$, or thin paths ($M=0.82,SE=0.03$), with $p<0.001$. Similarly, observers were significantly slower in solving mazes with medium paths compared to mazes with thin paths, with $p<0.01$.

#### Error rate

Error rate was defined as the proportion of trials where observers incorrectly identified solvable mazes as insolvable or vice versa. The chance level was 50% in each condition. Across all conditions, error rates were well below the chance (thick path: 1%, medium path: 2%, and thin path: 2%), suggesting that observers were highly accurate in their decisions. The differences in error rates between conditions were small and not significant ($p>0.05$).

### Discussion

The goal of Experiment 1 was to test the effect of path thickness on maze-solving performance. The results demonstrate that increasing path thickness decreases maze-solving efficiency. Next, we discuss the results in the context of the models of Jolicoeur et al. (1991) and Pooresmaeili and Roelfsema (2014).

Whether the models of Jolicoeur et al. (1991) and Pooresmaeili and Roelfsema (2014) explain the results of Experiment 1 depends on how one generalizes those models to stimuli more complex than thin lines on a blank background. We take as a fundamental property of the model that the size of the growth cone is expected to extend until some image-based constraint is encountered. To generalize the growth cone model to new stimulus classes, however, the nature of this constraint can be interpreted in different ways with different consequences for predicted performance. We propose two possible interpretations: The first possibility is that the growth cone would extend until encountering the edge of the flanking paths. This distance gets larger for thinner paths, correctly predicting faster completion times for the narrower paths in our mazes (see Fig.  7, blue circles, for an example). This interpretation of the growth cone model is consistent with its use on the [Roelfsema-like] stimuli of Fig. 4a.

However, this interpretation may not successfully generalize to mazes that include image complexity not directly related to the topology of the maze paths. For example, mazes in Fig. 3 include embellishments of the maze walls. If the constraint of the growth cone size is determined by the neighboring paths, the cone would contain considerable clutter (see Fig. 4b, blue circle) – the animal shapes, rocks, and stars in these decorative mazes. This outcome seems antithetical to the notion that these processing regions contain only the current path and no other “stuff”. While Experiment 1 did not include stimuli to test this proposal, we think it is a clear limitation of this interpretation of the growth cone model that would likely require additional parameters to specify how growth cone size is affected by such extraneous elements.

A second possibility is that the growth cone would extend outward from the center of the current path until encountering, say, a significant V1 response. For stimuli such as those in Jolicoeur et al. (1991) and Pooresmaeili and Roelfsema (2014), this could have the effect of extending to the neighboring paths, as before. For the complex mazes in Fig. 3, the regions might extend until encountering the walls, which has the benefit of limiting growth cone scale to portions of the maze design that are relevant to its topology. However, this strategy would also seem to predict larger growth cones for our thick path mazes than our medium path mazes. A medium path only affords expansion of the growth cone until it fills the narrower corridor, leading to the (incorrect) prediction that one could solve thick path mazes more quickly by virtue of the larger-scale growth cones that can expand to fill wider paths. Using something like V1 responses as the basis for constraining growth cone scale using an image-based criterion thus does not account for our data.

Alternatively, perhaps the visual system has two strategies for deploying growth cones: one for simple curve following and simple mazes, and another for more complex mazes. For the simple mazes in this experiment, it would allow the growth cones to continue at least until reaching the neighboring path (Fig. 7a, blue circles) – for such simple stimuli even with crowding it might be possible to process further along the path. For the complex mazes such a mechanism would detect the clutter and keep the growth cones smaller (Fig. 4b, red circle). The spacing-dependence in previous models was at least in part a stand-in for a model of crowding, back when none existed. More recent work has developed a high-performing model of crowding (Rosenholtz et al., 2019), which can deal with complex stimuli, and goes beyond thinking of crowding merely in terms of the spacing of stimuli. Experiment 1 does not provide a critical test of these competing accounts, but this discussion highlights the need to further understand how clutter unrelated to maze topology may constrain visual processing.

Experiment 2 provides insight into the impact of appearance changes on maze-solving performance. Predicting our data from Experiment 1 with growth cones would require that we elaborate on the basic model to suit our stimuli and task demands more closely. As such, we continue by investigating other changes in maze appearance that do not affect the maze topology, in Experiment 2, we constructed two sets of mazes with thick and medium path sizes, and varied the maze complexity by making the paths/walls of these mazes wavy in appearance, while keeping the path directions the same.

## Experiment 2

### Methods

#### Observers

A total of 24 observers participated in the experiment. All were naïve to the purpose of the experiment and received compensation for their participation. All observers self-reported normal or corrected-to-normal visual acuity.

#### Apparatus

We used the same apparatus described in Experiment 2.

#### Stimuli

**Fig. 8:**
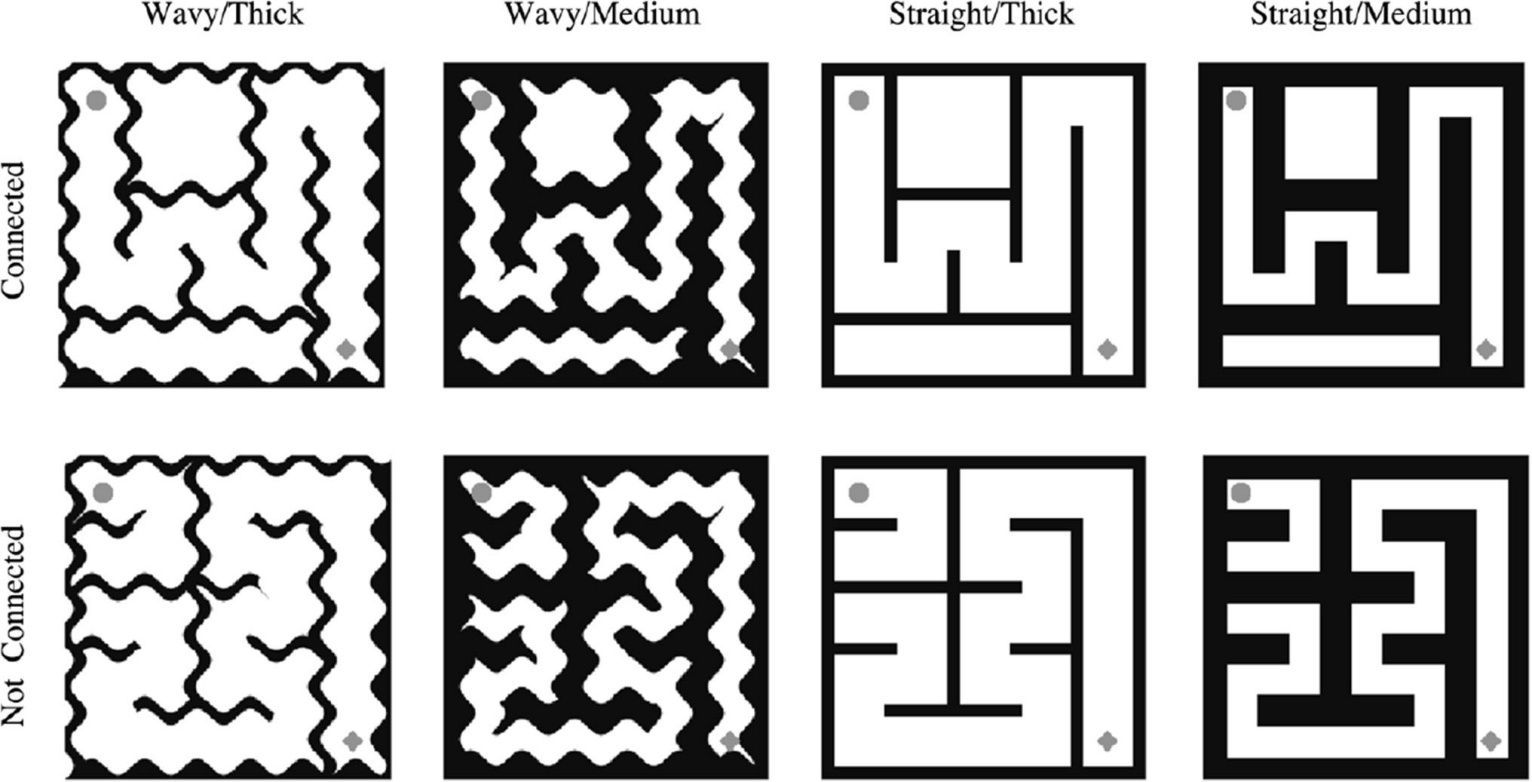
Example stimuli used in Experiment 2. *Columns left to right* show mazes with wavy walls and thick paths, wavy walls and medium paths, straight walls and thick paths, and straight walls and medium paths. *Rows top to bottom* show mazes with connected paths (top left and bottom right cells, marked by a *dot* and a *diamond*, are connected) and mazes with disconnected paths

Thirty $5 \times 5$ mazes were generated with Python using a maze generation algorithm and are all different from the mazes used in Experiment 2 (Fig. 8). Each maze was then flipped $180^{\circ }$ to create another maze, resulting a total of 60 mazes in our stimuli set. As in Experiment 2, half of the mazes had a path connecting the top left and bottom right cell, marked by a dot and a diamond, respectively. The other half did not have a path connecting the top left and bottom right cell. Each maze extended $8^{\circ } \times 8^{\circ }$ of visual angle.

We manipulated the path thickness (medium or thick) and path style (straight or wavy, 1 cycle/unit). The path width was set to $0.8^{\circ }$ and $1.3^{\circ }$ of visual angle in the medium and thick path conditions, respectively. The straight mazes were modified to be wavy using Adobe Photoshop 20 (Adobe, San Jose, CA, USA) software.

#### Procedure

The task and the procedure were the same as in Experiment 2. Mazes were blocked by the path thickness and path style. Each participant completed eight blocks with 30 trials in each block, resulting a total of 240 trials. The block sequence was semi-randomized across participants.

### Results

#### Response time

**Fig. 9:**
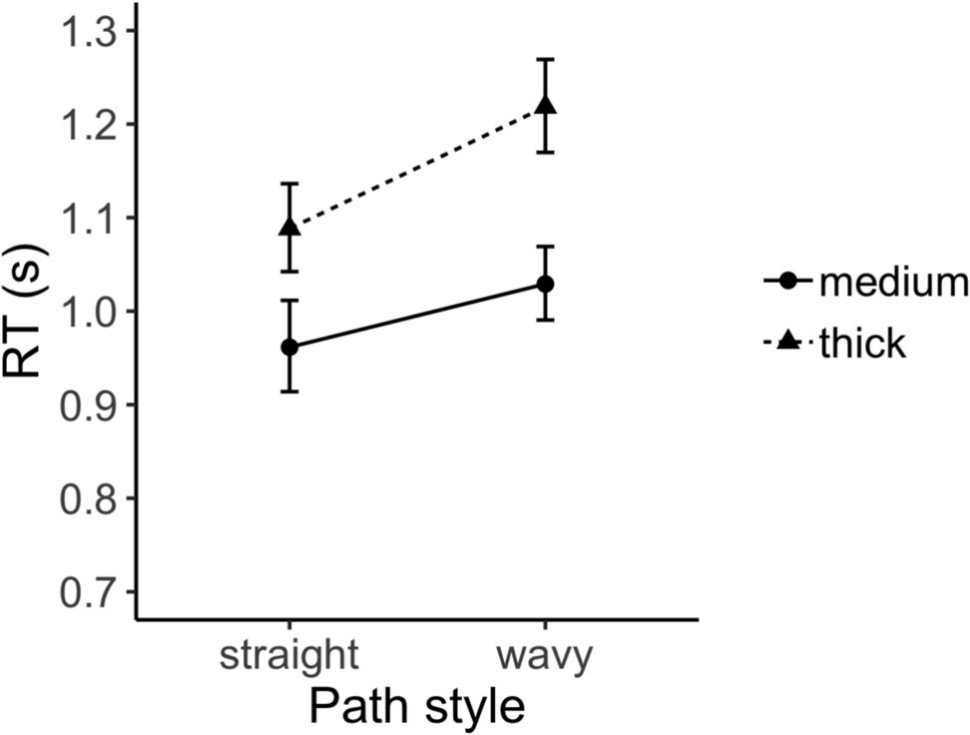
Average response times (s) in each condition (back-transformed from log for easier interpretation). As the thickness of the path decreases, so does the response time. Also, the time required to solve wavy mazes is longer than the time required to solve straight mazes. *Error bars* indicate standard error of the mean

We analyzed log-transformed response times for correct trials. Figure 9 shows mean of these response times in the straight and wavy mazes with medium and thick paths (back-transformed from log for easier interpretation). A two-way repeated measures ANOVA, with path style (straight or wavy) and path thickness (medium or thick) as within-subjects variables. The ANOVA revealed main effects of path style ($F(1,23)=19.85,p<0.001, \eta ^2=0.46$) and of path thickness ($F(1,23)=23.51,p<0.001, \eta ^2=0.51$). In particular, observers were slower while solving the wavy mazes ($M=1.12,SE=0.03$) than the straight mazes ($M=1.02,SE=0.03$). In addition, observers were slower while solving the mazes with thick paths ($M=1.15,SE=0.03$) than the mazes with medium paths ($M=0.99,SE=0.03$). The interaction effect was not significant ($F(1,23)=2.29,p<0.05$).

#### Error rate

As in Experiment 2, error rate was calculated as the proportion of trials where observers incorrectly identified solvable mazes as insolvable or vice versa and the chance level was 50% for each condition. Across all conditions, error rates were well below chance (straight mazes: 2% both in thick and medium paths, wavy mazes: 3% both in thick and medium paths), suggesting that observers were highly accurate in their decisions. There were no significant differences between conditions in terms of error rates ($p>0.05$).

### Discussion

The results of Experiment 2 show that maze-solving time is longer for mazes with wavy walls than mazes with straight walls. Furthermore, we see the same pattern of results as in Experiment 1, with thicker paths leading to a slower solution than medium paths. Taken together, these findings suggest that the appearance of the maze walls clearly matters. Visual complexity in mazes increase maze-solving difficulty. Next, we discuss the results in the context of the models of Jolicoeur et al. (1991) and Pooresmaeili and Roelfsema (2014).

**Fig. 10:**
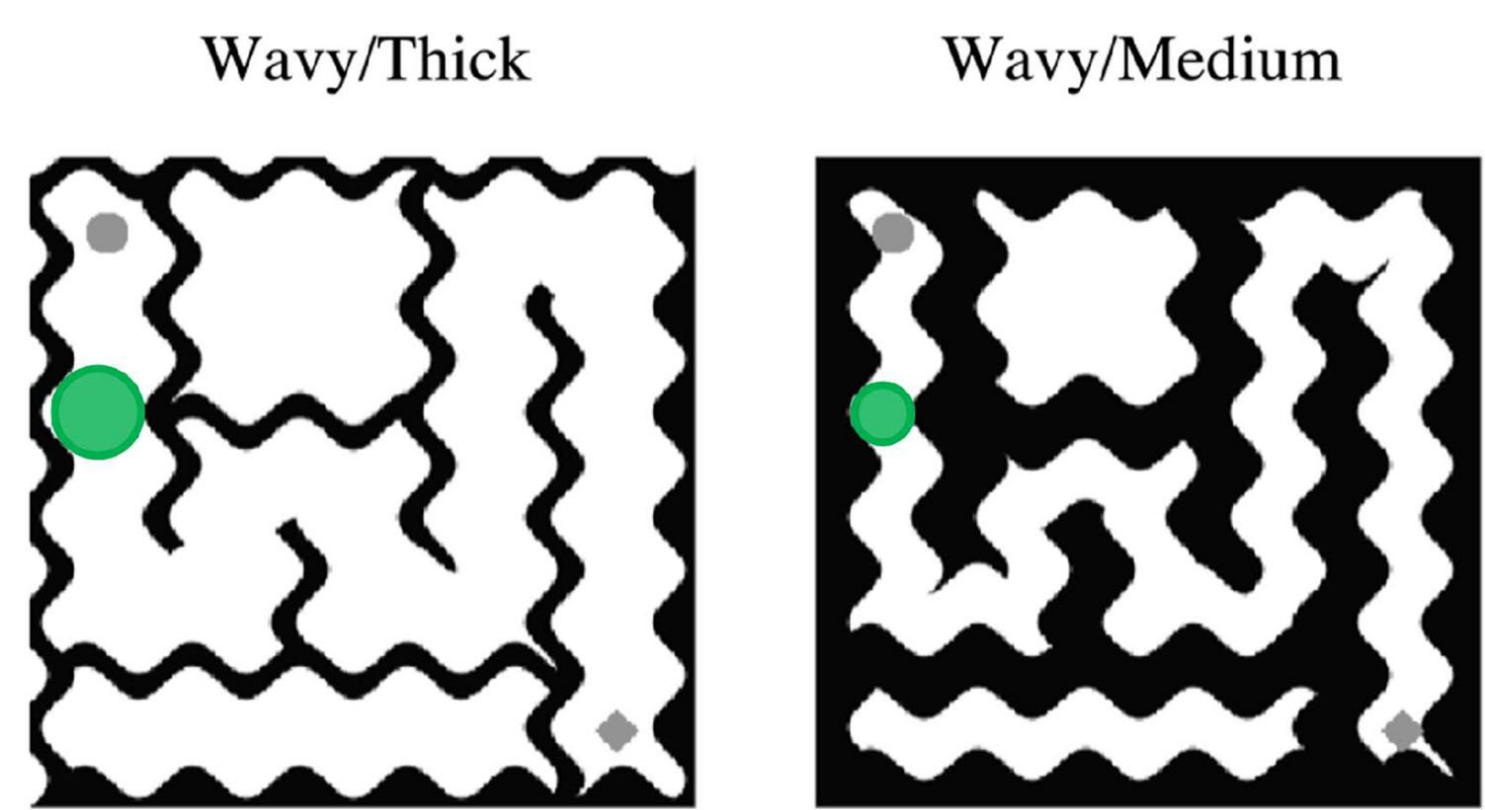
A simple but more flexible interpretation of the growth cone model might have two modes, adjusting cone size based on the stimulus. For uncluttered stimuli the growth cone might extend to the neighboring path (Figs. 4a, and 7, *blue circles*). For more cluttered stimuli of Experiment 2, the flexible model might restrict the growth cone to the path (shown here in *green*). The cone size is bigger for the wavy/thick condition than for the wavy/medium condition, predicting that the performance should be better for wavy/thick condition

To test whether a simple change to the growth cone model would suffice to generalize to more complex and cluttered stimuli, we asked whether adding a different mode, in which detection of cluttered stimuli led to smaller growth cones restricted to the path could explain results for the mazes with wavy walls and paths. Presumably, the two-mode model would decide the wavy mazes were cluttered and the growth cone should be restricted to the path. Figure 10 shows example growth cones for a flexible two-mode version of the growth cone model, on sample stimuli from Experiment 2. The cone size is bigger for the wavy/thick condition than for the wavy/medium condition, predicting that the performance should be better for wavy/thick condition. Contrary to these predictions, participants were faster to solve the wavy/medium mazes than the wavy/thick ones.

Peripheral crowding depends in complex ways on the exact details of the stimulus, and questioning a simple two-mode model does not eliminate a more intelligently flexible model that processes larger or smaller regions based on the degree of local crowding. We discuss this and other possible factors in the *General discussion* section.

Experiment 2 manipulated visual complexity in a basic way that was easy to implement as a transformation on existing mazes. One would of course like to explore alternative ways of varying the visual complexity of mazes while controlling factors like path length and number of turns. In addition to texturing walls out of caterpillars, letters, or animals, one might also texture the paths with, for instance, gravel. Our experiment is only the initial step at tackling a complex problem.

## General discussion

Efficient maze solving requires observers to get the gist of the path ahead rather than relying purely on local or foveal information. This process necessarily involves peripheral vision, which allows observers to perceive the path beyond the current fixation (Jolicoeur et al., 1986). If so, we would expect crowding, the dominant effect in peripheral vision, to play a role in maze-solving performance. Crowding is a stimulus-specific effect and depends heavily and in a complex way on the design of the stimulus, and not merely on the path density. Under conditions of crowding, processing peripheral stimuli becomes more difficult, but considerable information can remain for the observer potentially to view the shape of the path ahead, keep track of the exit, distinguish between wall and path, and extract figure–ground relations (Rosenholtz, 2016). Our goal was to investigate the role of crowding and visual complexity in mental maze solving by examining the effects of perceptual aspects of maze design on maze-solving performance. Using controlled stimuli, we examined the effects of thickness of the paths as well as style of rendering the paths and walls between them. Experiment 1 found that mazes with thinner paths required less time to solve compared to mazes with thicker paths. Experiment 2 replicated the effect of path thickness and showed that mazes with wavy walls, which are likely more crowded, require more time to solve compared to mazes with straight walls. These results contribute to the literature by (1) showing manipulations that should affect performance based on known properties of crowding play a role in a complex task, while (2) also evaluating how well a competing model (i.e., the growth code model) can explain the findings.

Up to this point we have mostly framed the experiments and results in terms of the growth cone model of Pooresmaeili and Roelfsema (2014). As described in the Introduction, the dependence of visual cognition tasks on clutter has led a number of researchers to draw connections between such tasks and peripheral crowding. It is worth some discussion of what a peripheral vision model of maze solving might look like, particularly so as to clarify connections to the growth cone model. The dominant model of peripheral crowding (Rosenholtz, 2016) suggests that crowding arises due to computation of summary statistics over “pooling regions” that grow in size as a linear function of eccentricity. It would be tempting, but a misconception, to directly equate these pooling regions with growth cones. A growth cone is a *mechanism* that varies in size in response to the stimulus, whereas pooling regions are fixed in size at a given location in the visual field, independent of the stimulus. (Rosenholtz et al. (2019) discuss issues with alternative suggestions of flexible pooling regions.) What does vary with the stimulus is the region over which peripheral vision does a good job of representing task-relevant visual information; essentially the “functional visual field” (Young & Hulleman, 2013) over which peripheral vision provides largely unambiguous information for one’s task. The functional visual field is not a mechanism but a *result* of peripheral mechanisms. For simple paths, fixed peripheral pooling might provide clarity over a long stretch of path, leading to fast solution times. For more cluttered paths, peripheral vision would provide unambiguous information over a shorter distance, after which in normal maze solving the participant would move their eyes to gather more information. Future work is necessary to distinguish between a peripheral vision explanation and other alternatives ways in which stimulus complexity might affect maze solving.

The dependence of performance on path thickness may result from greater crowding for thick as opposed to thin paths. It is unclear whether earlier models can predict this result, and reasoning about diverse mazes has pointed to a need to clarify those models when applied to a broader set of stimuli. Another possibility, which we favor, is that the effect of path thickness for these mazes depends on the Gestalt principle of figure–ground. A number of perceptual cues have been identified which guide observers’ decision in figure–ground segmentation (for a review, see Wagemans et al. (2012)). “Configural cues" (i.e., small area, closure, convexity, and symmetry) can be used to identify the figure as opposed to the ground (Harrower, 1936; Peterson & Skow-Grant, 2003; Rubin, 1915). A number of these factors are not well-defined for paths and walls in simple mazes (let alone complex mazes that incorporate illustration elements) or are not likely to be factors in maze design. Symmetry, for example, can be a feature of maze appearance but is certainly not guaranteed to be a property of path or wall layout. The impact of other appearance manipulations, our waviness manipulation in particular, are not straightforward to evaluate in terms of strengthening or weakening figure–ground assignment. Small-area, however, may be relevant to the results we observed with thin paths insofar as observers may more easily identify thinner maze paths as figure, which likely facilitates perceiving the shape of those paths (Peterson & Enns, 2005). The association with figure–ground processing, as well as our understanding of the complexity of visual crowding phenomena, suggest caution in generalizing our path thickness results to more complex mazes such as those in Fig. 3. This need for caution is complemented by intriguing possible directions for further work to examine the relationship between crowding and figure–ground segmentation in path-finding and connectedness judgments closely.

The diversity of maze designs suggests a rich domain of future study. For example, in some of the children’s mazes in Fig. 3, it may be more difficult for the observer to judge what is wall vs. path than in our simpler mazes in our experiments, particularly in the periphery. This might make maze solving slower, for example by forcing observers to make shorter saccades along the path in order to distinguish path from wall. Some of the maze designs may make it harder to tell the location of gaps in the walls in the periphery. The paths and walls of the various mazes seem subjectively to have different amounts of clutter, which could affect crowding and performance. In fact, the design of the maze can result in an interaction effect between some of these possible factors: on the one hand, mazes with uncluttered paths and walls might be easy to solve because of diminished crowding, but hard to solve because of the lack of texture clearly distinguishing the path from walls.

Moving forward, a number of approaches can lend insight into mental maze solving and aid in the development of new models. First, additional studies monitoring fixation can potentially help our understanding of what critical points in the mazes require more study in order to plan the path ahead. Controlled experiments in which observers must fixate and answer questions about the path ahead can aid in determining the degree to which performance depends on peripheral vision. For instance, one might ask about ability to identify the shape or extent of the path ahead, or to localize portions of that path. Indeed, studies of visual crowding (e.g., Bouma, 1970; Levi, 2008; Pelli et al., 2004, 2007) and peripheral position uncertainty (e.g., Michel and Geisler, 2011; Pelli, 1985; Semizer and Michel, 2017; Tanner, 1961) suggest that observers’ ability to identify and localize features declines in the visual periphery. One might ask whether, for instance, the wavy or more cluttered mazes in our stimuli set impair observers ability to see further along the path, all else being roughly equal. In the process, we can potentially use this rich and relatively easily controlled stimulus domain to learn about a number of aspects of vision science, from peripheral crowding, through eye movement planning, to visual cognition tasks and visual routines.

## Data Availability

Data are available upon request from the corresponding author. None of the experiments were preregistered.

## Footnotes

1 The notion of a “processing region” suggests an attended region, which Pooresmaeili and Roelfsema (2014) make explicit. Though the question of the role of attention in maze solving speaks to the degree to which mechanisms operate serially as opposed to in parallel, it is out of scope of the present paper.

2 Three participants viewed stimulus on a 17-in Dell 1704 FRV display at a viewing distance of approximately 50 cm. Stimuli are scaled to the same visual angle as on the other setup.
